# Advances in traditional Chinese medicine for burn treatment: mechanisms, therapeutic approaches, and innovative preparations

**DOI:** 10.3389/fphar.2025.1651219

**Published:** 2025-11-17

**Authors:** Ying Liu, Caiyuan Yu, Tong Dou, Yang Shen, He Li, Mengyan Liu, Wenying Xie, Weijia Zeng, Zizhao Feng, Ming Huang, Yizhun Zhu

**Affiliations:** 1 School of Pharmacy, Faculty of Medicine, Macau University of Science and Technology, Taipa, Macao SAR, China; 2 School of Pharmacy, Guangzhou Xinhua University, Guangzhou, China; 3 School of Pharmacy, Sun Yat-sen University, Guangzhou, China

**Keywords:** traditional Chinese medicine, burn injury, active ingredients, mechanism, preparation

## Abstract

Traditional Chinese Medicine (TCM) possesses a well-documented historical legacy and substantial clinical experience in treating burn injuries and diverse wound conditions. Grounded in TCM theory, therapeutic strategies incorporate herbal medicine and its external preparations, as well as TCM auxiliary treatment, forming a comprehensive treatment framework. A systematic evaluation of burn management, particularly recent advancements in TCM research, carries significant implications for both theoretical and clinical applications. This paper synthesizes information from a plethora of online resources to explicate the mechanisms of TCM in burn treatment from multifaceted perspectives. Specifically, a comprehensive collection of literature pertaining to TCM burn treatment from the past three decades was amassed from electronic databases including PubMed, CNKI, and Web of Science. A meticulous keyword information statistical analysis was performed on this corpus. The search strategy employed keyword clusters such as “traditional Chinese medicine, phytochemistry, or herbs” combined with “burn, scald, or skin wound”. The scientific nomenclature of plants was verified using “The Plant List” (www.plantsoftheworldonline.org). This review encapsulates the methodologies of burn treatment within TCM and underscores a multitude of herbs with burn-treating capabilities, including *Arnebia euchroma* (Royle ex Benth.) I.M.Johnst., *Rheum palmatum* L.*, Coptis chinensis* Franch., *Phellodendron chinense* C.K.Schneid., *Sanguisorba officinalis* L., and *Angelica sinensis* (Oliv.) Diels*,* as well as natural borneol (from *Dryobalanops aromatica* C.F.Gaertn.), Frankincense (from *Boswellia sacra* Flück.), and Myrrh (from *Commiphora myrrha* (T.Nees) Engl.). The principal active ingredients identified are shikonin, emodin, berberine, ferulic acid, and curcumin; however, their mechanisms warrant further in-depth investigation. Notable strides have been made in the innovation and research of TCM in burn treatment. Beyond traditional external formulations, hydrogel, liposome, microsphere, and nanofibers have emerged as pivotal elements in burn management. These advanced materials have introduced an innovative drug delivery system by integrating the active components, thereby enhancing the efficacy of burn treatment.

## Graphical Abstract

**Figure:**
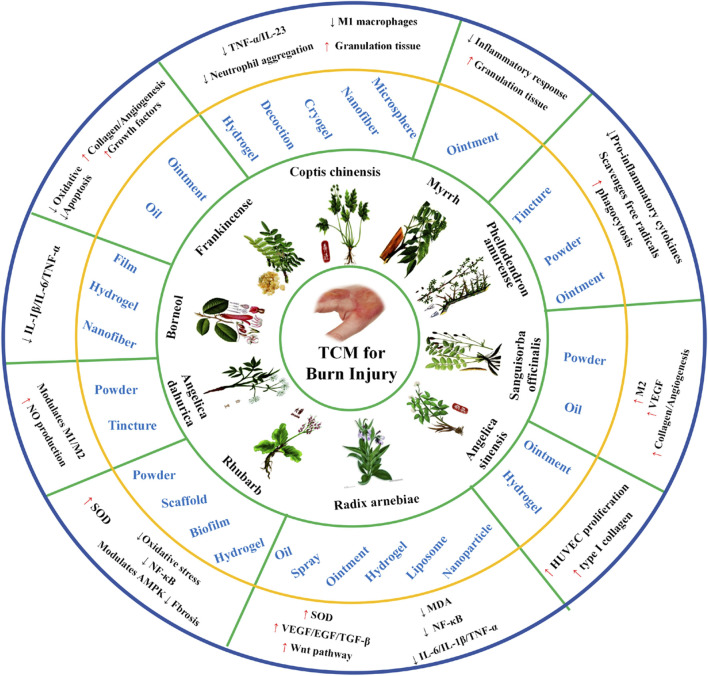

## Introduction

Burns represent a prevalent form of accidental injury in clinical practice, with an estimated 70 million cases occurring globally each year. These injuries result in approximately 18 million disabilities and over 20,000 deaths annually (Peck, 2011; 2012). Burns are caused by thermal, electrical, radiation, chemical (acids, alkalis, irritants, and corrosive substances), or other physical and chemical factors, leading to damage and necrosis of superficial and subcutaneous tissues, accompanied by a cascade of pathological changes (Jeschke et al., 2020). The efficacy of burn treatment depends on the extent and severity of the injury, as well as the timeliness and precision of therapeutic interventions (Radzikowska-Büchner et al., 2023). Conventional treatments often include antibiotics, energy supplements, micronutrients, immune-modulating agents, and topical growth factors or recombinant human growth hormone therapy. However, these treatments may carry potential side effects, such as disruptions to metabolic processes, induction of secondary diseases, or drug dependence (Roshangar et al., 2019; Markiewicz-Gospodarek et al., 2022).

Traditional Chinese Medicine (TCM) has a long history of treating burns, with the earliest records dating back to the *Fifty-Two Diseases Prescriptions* from the pre-Qin period in recent years, TCM has gained increasing recognition in burn management due to its notable efficacy, low toxicity, and diverse formulation types (Zhou et al., 2024). Despite this, there is a notable lack of comprehensive reviews on TCM approaches to burn treatment. Despite this, there is a notable lack of comprehensive reviews on TCM approaches to burn treatment. The existing literature on this topic often remains at the overview level (Kopp et al., 2003; Herman and Herman, 2020; Mrabti et al., 2022), and more detailed studies exploring the underlying mechanisms are still needed. Furthermore, reviews that integrate modern medical methodologies with TCM strategies or explore burn treatment from a contemporary TCM perspective are currently limited.

To address this research gap, a systematic and comprehensive literature review was conducted to evaluate the current application of TCM in burn treatment. The literature search was performed across multiple electronic databases, including PubMed, CNKI, and Web of Science. The search strategy utilized the following keyword clusters: (“traditional Chinese medicine” OR “phytochemistry” OR “herbs”) AND (“burn” OR “scald” OR “skin wound”). The scientific nomenclature of medicinal plants was verified using “The Plant List” (www.plantsoftheworldonline.org). The initial search yielded 92 records from PubMed, 256,153 from CNKI, and an unspecified number from Web of Science. After importing all records into EndNote literature management software (https://endnote.com/), 75 duplicates were removed. The remaining articles underwent a two-stage screening process based on the following criteria: Inclusion Criteria: Studies focused on the use of TCM compounds, single herbs, or active ingredients in the treatment of burns, scalds, or skin wounds. Articles published in peer-reviewed journals within the past 30 years. Reports available in either English or Chinese. Studies involving *in vitro*, *in vivo*, or clinical evaluations. Exclusion Criteria: Publications not relevant to TCM or burn treatment (e.g., veterinary use, non-therapeutic research). Non-original research articles such as editorials, commentaries, or conference abstracts without full text.

**FIGURE 1:**
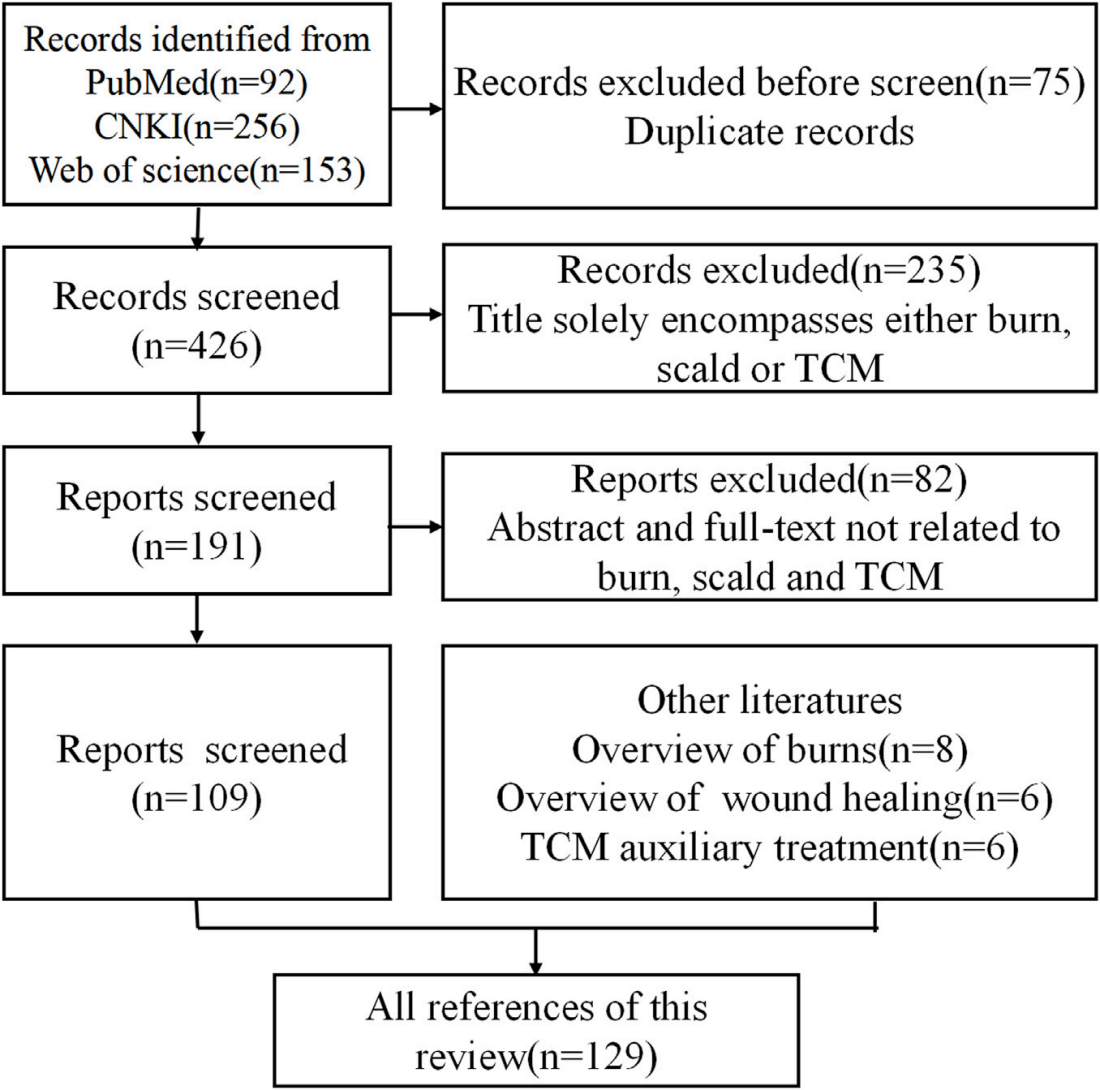
Flowchart for inclusion and exclusion of literature in this review.

After title and abstract screening, irrelevant studies were excluded manually. Ultimately, 109 articles met the inclusion criteria and were selected for in-depth analysis. Additionally, 20 supplementary literatures were included to provide foundational context: 8 pertaining to general burn overviews, 6 related to wound healing mechanisms, and 6 focused on TCM auxiliary therapies. The literature selection process is summarized in Figure 1.

This paper summarizes the understanding of burns in TCM and modern medicine, particularly focused on underlying mechanisms, therapeutic approaches, innovative external preparations, and TCM auxiliary therapies, aims to provide valuable insights and research directions regarding therapeutic drugs for burn management.

### Burn pathology in modern medicine

Burn injuries are classified into four degrees (I–IV) based on the depth and extent of tissue damage (Warby and Maani, 2023). Superficial burns (first-degree or superficial second-degree) affect only the epidermis, typically healing with minimal scarring. Deep second-degree burns involve the deep dermal layer, presenting with blisters, a red-white base, and increased exudate, often leading to hypertrophic scarring. Third-degree burns extend through the entire dermis, usually requiring surgical intervention, while fourth-degree burns involve deeper structures such as muscle or bone, resulting in significant functional impairment (Żwierełło et al., 2023).

**FIGURE 2:**
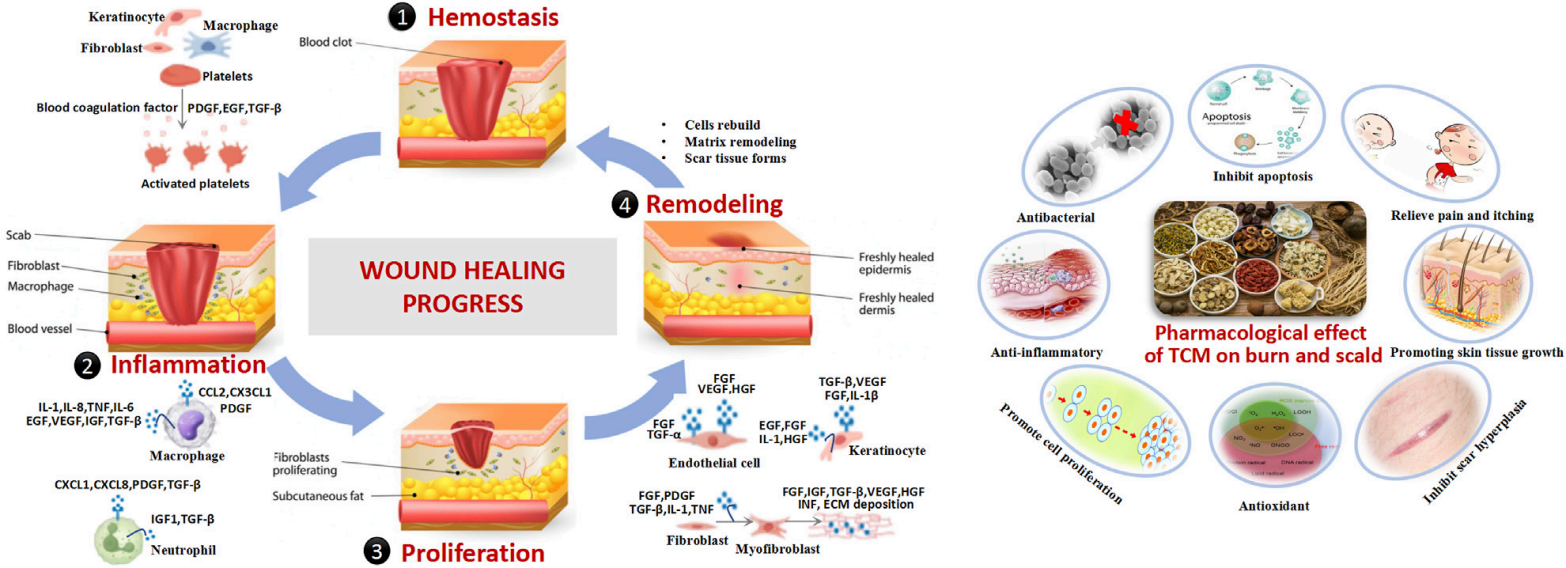
Burn pathology in modern medicine.

Wound healing aims to restore tissue integrity and homeostasis through three overlapping phases: inflammation, proliferation, and remodeling. Disruptions in these phases can lead to delayed healing or chronic wounds (Wallace et al., 2023; Pena and Martin, 2024). Inflammatory Phase initiated by immune responses to remove damaged tissue and pathogens, this phase involves vascular and cellular responses. Cytokines such as transforming growth factor (TGF-β), platelet-derived growth factor (PDGF), epidermal growth factor (EGF), fibroblast growth factor (FGF), and interleukin-8 (IL-8) are released, recruiting neutrophils and macrophages for pathogen clearance, inflammation resolution, and tissue repair (Childs and Murthy, 2017; Wilkinson and Hardman, 2020). The proliferative phase occurs 2–10 days post-injury, this phase is driven by macrophages, which release growth factors to recruit fibroblasts and keratinocytes. Angiogenesis, mediated by vascular endothelial growth factor (VEGF) and FGF, is initiated, and fibroblasts synthesize extracellular matrix (ECM) proteins to form granulation tissue (G El Baassiri et al., 2023). Finally, remodeling phase begins 2–3 weeks post-injury and lasting up to 2 years, this phase involves the maturation of the scar. Fibroblasts, macrophages, and endothelial cells secrete matrix metalloproteinases (MMPs) to degrade type III collagen, which is replaced by type I collagen organized into parallel fibrils. Apoptosis of excess cells and remodeling of the epidermis, vasculature, and nerves occur during this stage (Mathew-Steiner et al., 2021). Effective wound healing relies on growth factors, nutrient supply, cell-to-cell interactions, and oxygen availability. Disruptions due to infection, malnutrition, chronic diseases, or diabetes can impair healing, leading to chronic wounds. Understanding these mechanisms is critical for optimizing burn treatment strategies (Figure 2).

### TCM perspective on burn

**FIGURE 3:**
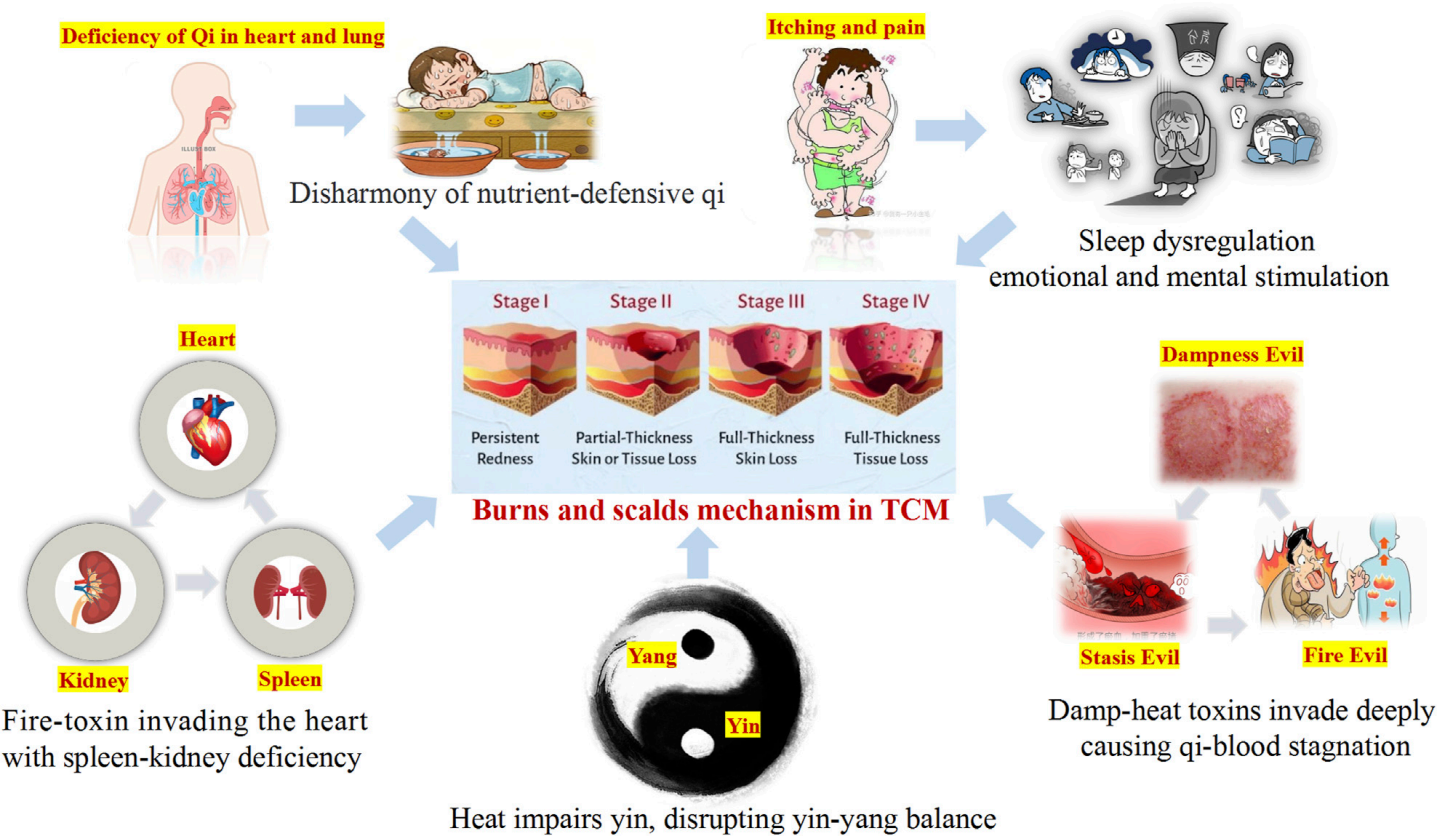
TCM perspective on burn injury.

In TCM, burn injury is collectively referred to as “water and fire burns”. It occurs when the skin and deeper tissues are exposed to high temperatures, leading to Qi and blood stagnation and an imbalance between defensive (Wei) Qi and nourishing (Ying) Qi. The pathogenesis involves heat acting on the superficial muscles, causing local Qi stagnation and meridian obstruction. This weakens the Wei Qi, the body’s first line of defense, reducing its protective function and resulting in fluid leakage, blister formation, and exudate (Que et al., 2005). Excessive blistering may deplete Yin fluids, eventually leading to Yin deficiency, Yang collapse, and an imbalance between Yin and Yang (Ripszky Totan et al., 2022). Furthermore, the invasion of fire toxins can impair the functions of the spleen, kidney, and heart, exacerbating Qi stagnation. Sleep disorders in burn patients are closely associated with Yin-Yang imbalances and internal organ dysfunctions (Liang et al., 2021; Liu et al., 2023). In TCM, normal sleep relies on the harmonious coordination of Yin-Yang and mental tranquility. Disruptions in these factors or mental unrest can lead to sleep disturbances (O'Brien and Weber, 2016). Burn patients often experience severe nocturnal skin itching, which disrupts sleep and may result in neurasthenia or other health complications (Chung et al., 2020) (Figure 3).

Based on the fundamental tenets of TCM, burn injuries can be classified into three distinct stages according to their typical symptomatic manifestations: the stage of excessive heat - toxin accumulation, the stage of Yin - fluid depletion, and the stage of Yin deficiency. These stages often exhibit overlapping pathological features. In the early stage, treatment predominantly focuses on heat-clearing and detoxification strategies to prevent the intrusion of toxins into the body. In the middle stage, the emphasis lies in nourishing Yin, promoting blood generation, clearing heat toxins, and facilitating tissue regeneration. In the late stage, the focus is on Qi supplementation, blood nourishing, and regulating the balance of Qi and blood.

### Treatment of burn with Chinese herbal medicine

**FIGURE 4:**
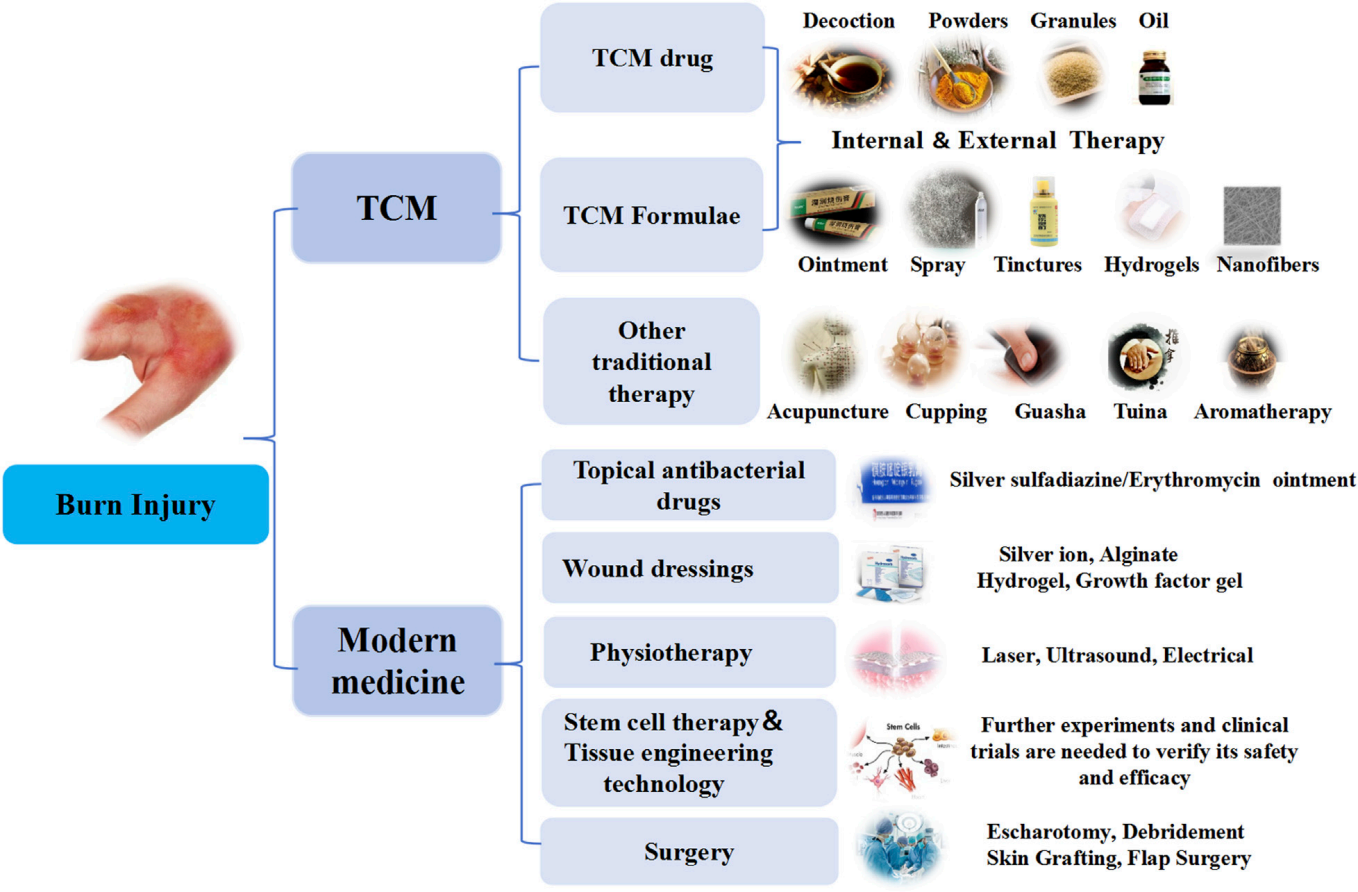
Treatment of burn injury with TCM and modern medical.

With the advancement of modern TCM, there is a growing body of theoretical and practical evidence supporting its use in burn treatment. TCM employs a holistic approach, utilizing single herbs (e.g., *Rheum palmatum* L.), *Coptis chinensis* Franch., *Angelica sinensis* (Oliv.) Diels) and compound prescriptions to address both systemic and local symptoms of burn injuries. Therapeutic methods include internal administration, which regulates systemic conditions and enhances the body’s resistance to pathogens, and external application, which directly targets the burn site to promote wound healing and prevent complications such as tissue necrosis, vascular occlusion, and infection. External medications, including ointments, sprays, and hydrogels, play a critical role in addressing local blood circulation disorders and controlling wound infections. Additionally, auxiliary TCM therapies such as acupuncture, cupping, guasha, tuina, and aromatherapy provide complementary benefits by improving blood flow, reducing pain, and enhancing overall recovery (Figure 4).

Chinese herbal medicine and natural products are widely recognized for their efficacy, low side effects, and minimal resistance development in treating burns. The healing process of burn wounds is highly complex, involving growth factors, inflammatory mediators, and ECM remodeling. TCM exerts its therapeutic effects through multi-component, multi-target, and multi-pathway mechanisms. Experimental studies have demonstrated that products used in TCM can enhance the production and secretion of growth factors, including VEGF and EGF. Additionally, they have been shown to reduce the expression of pro-inflammatory cytokines such as interleukin-6 (IL-6) and tumor necrosis factor-alpha (TNF-α), inhibit bacterial proliferation, improve immune function, and regulate collagen synthesis along with ECM remodeling. Furthermore, TCM-based treatments exhibit antioxidant properties, eliminate free radicals, facilitate cell proliferation, inhibit apoptosis, and provide analgesic and antipruritic effects.

This review highlights the active ingredients, traditional uses, pharmacological actions, target functions, and mechanisms of commonly used Chinese herbal medicine, underscoring their potential in modern burn treatment strategies (Table 1).

**TABLE 1:** Commonly used Chinese herbal medicine for treating burns.

Chinese herbal medicine	Origin and medicinal parts	Active ingredients	Traditional use	Pharmacological action	Target function	Mechanism	References
Radix arnebiae (Zi Cao)	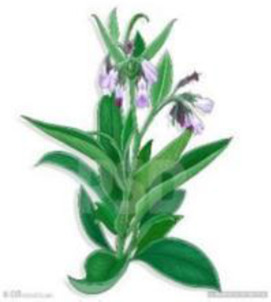 *Arnebia euchroma* (Royle) Johnst Rhizoma	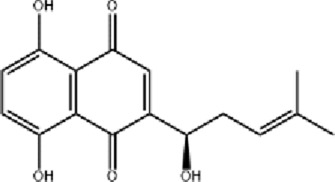 Shikonin	Cool blood and promote blood circulation Clear internal heat Neutralize toxins	Anti-inflammatory Wound healing Promotes epithelial regeneration	TGF-β1/PI3K/Akt NF-κB Wnt4 Bax/Bcl-2	↑SOD activity ↓MDA ↓IL-6/IL-1β/TNF-α ↑VEGF/EGF/TGF-β Activates Wnt pathway Inhibits NF-κB	Shu et al. (2022), Sun et al. (2022), Wu T. et al. (2022), Gao et al. (2023)
Rhubarb (Da Huang)	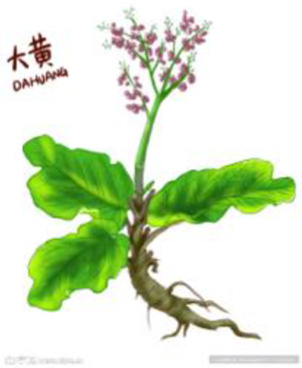 *Rheum palmatum* L. Rhizoma	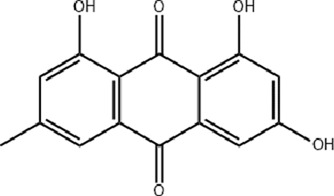 Emodin Rhubarb-derived characoal	Eliminate heat toxins Remove accumulations and stagnations Promote circulation of blood stasis	Anti-inflammatory Antimicrobial Promotes collagen synthesis	TLR4/NF-κB AMPK/mTOR Notch/TGF-β	↑SOD ↓oxidative stress Inhibits NF-κB Modulates AMPK pathway to reduce fibrosis	Tang et al. (2007), Sánchez et al. (2020), Wang et al. (2023), Tan et al. (2024)
Angelica sinensis (Dang Gui)	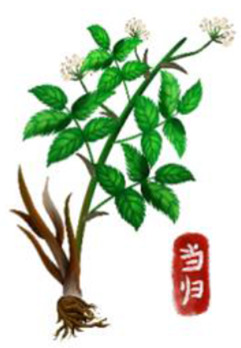 *Angelica sinensis* (Oliv.) Diels Root	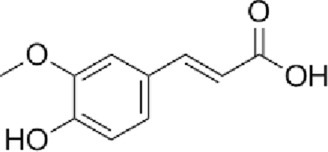 Ferulic acid Polysaccharides	Blood-nourishing and circulation-activating Menstruation-regulating and pain-relieving Intestine-moistening and laxative	Anti-inflammatory Angiogenesis Collagen synthesis Promotes re-epithelialization	p38/JNK1/2 VEGF	↑HUVEC proliferation ↑type I collagen	Lam et al. (2008), Zhao et al. (2012), Tsai et al. (2016), Li et al. (2021)
Coptis chinensis (Huang Lian)	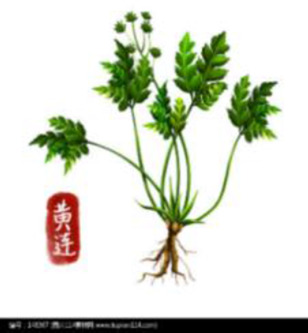 *Coptis chinensis* Franch Rhizoma	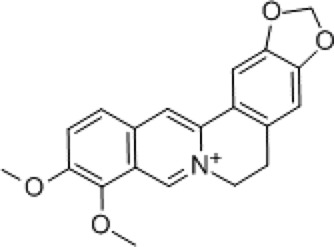 Berberine	Dispel pathogenic fire Remove dampness Neutralize toxins	Antibacterial Anti-inflammatory	NF-κB S100B/caspase-8/ β-catenin	↓TNF-α/IL-23 ↓neutrophil aggregation Enhance granulation tissue Inhibit M1 macrophages	Habtemariam (2020), Feng et al. (2024), Wang S. et al. (2024)
Phellodendron amurense (Huang Bo)	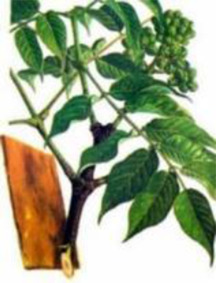 *Phellodendron chinense* Schneid. Bark	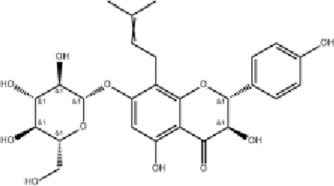 Phellamurin Berberine	Clearing internal heat Removing dampness Reducing pathogenic fire Detoxification	Immunomodulation Antimicrobial	INF-γ IL-1 TNF-α IL-2	↓Pro-inflammatory cytokines Scavenges free radicals Enhances phagocytosis	Xian et al. (2011), Liu et al. (2022)
Sanguisorba officinalis (Di Yu)	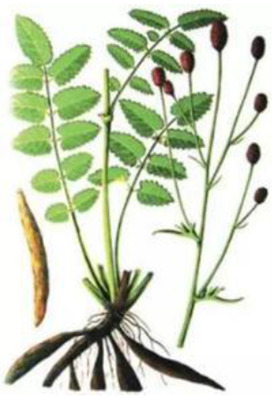 *Sanguisorba officinalis* L. Rhizoma	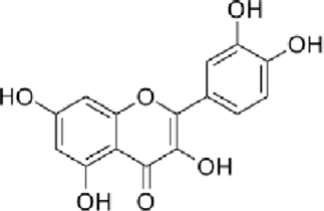 Quercetin Tannins Polysaccharides	Cool blood for hemostasis Polysaccharides Clear heat and remove toxins Reduce swelling Promote ulcer healing.	Antibacterial Anti-inflammatory	NF-κB/NLRP3 VEGF IL-1β	↓*S. aureus*/*P. aeruginosa* ↑collagen/angiogenesis Promotes M2 macrophage polarization	Zhang et al. (2018), Song et al. (2023)
Angelica dahurica (Bai Zhi)	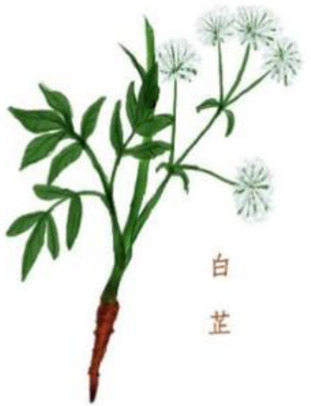 *Angelica dahurica (*Fisch.ex Hoffm.)Benth. et Hook.f. Rhizoma	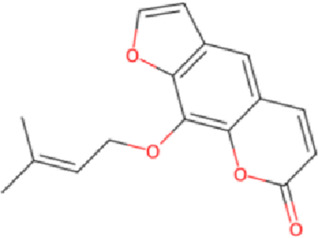 Imperatorin Isorhamnetin	Dispel pathogenic wind Eliminate dampness Subside swelling Relieve pain	Antibacterial Angiogenesis Anti-inflammatory	HIF-1α/PDGF-β ERK1/2/Akt/eNOS	Modulates M1/M2 macrophages ↑NO production	Zhang et al. (2017), Guo et al. (2020), Yang et al. (2020), Hu et al. (2021)
Natural Borneol (Long Nao)	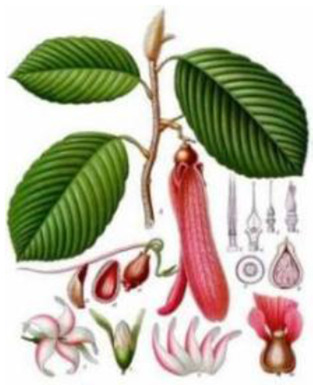 *Dryobalanops aromatica* C.F.Gaertn. Natural crystalline	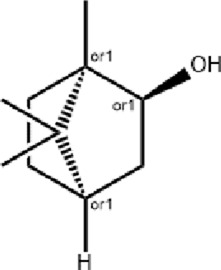 Borneol	Revives consciousness Reduces fever and pain Enhances vision and clears eye opacity	Antioxidant Anti-inflammatory Enhance collagen density	HIF-1α/NF-κB	↓IL-1β/IL-6/TNF-α	Barreto et al. (2016), Lv et al. (2022), Chen et al. (2024)
Frankincense (Ru Xiang)	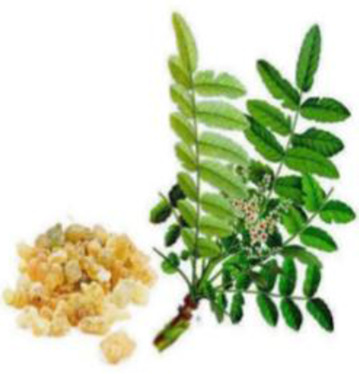 *Boswellia carterii* Birdw. Resin	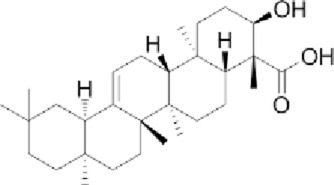 Boswellic acid	Regulate qi and promote blood circulation Relieve pain Eliminate toxins	Anti-inflammatory Tissue regeneration	β-catenin Dlk1 COX-2	↓Oxidative stress ↓Apoptosis ↑collagen/angiogenesis ↑Growth factors	Pengzong et al. (2019), Yin et al. (2022)
Myrrh (Mo Yao)	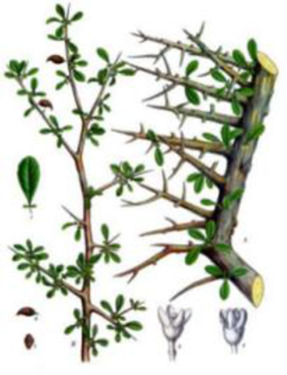 *Commiphora myrrha* (T.Nees) Engl. Resin	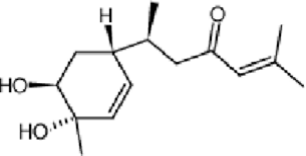 Bisacurone	Disperse and eliminate blood stasis Subside swelling Relieve pain	Angiogenesis Anti-inflammatory	Oxidative stress markers pro-inflammatory cytokines	↓Inflammatory response ↑granulation tissue	Soliman et al. (2019), Batiha et al. (2023), Yan et al. (2023)

## Radix arnebiae

Radix Arnebiae (Zi Cao), derived from the dried roots of *Arnebia euchroma* (Royle) Johnst. or *Arnebia guttata* Bunge as specified in the Chinese Pharmacopoeia, is widely used in TCM to clear heat, cool blood, promote circulation, detoxify, and eliminate rashes (Zhan et al., 2015). Clinically, it is often combined with herbs such as *Phellodendron amurense* Rupr.*, Saposhnikovia divaricata* (Turcz. ex Ledeb.) Schischk.*, Angelica dahurica* (Hoffm.) Benth. & Hook.f.*, Angelica sinensis* (Oliv.) Diels, and borneol to accelerate wound healing and reduce treatment time for burns (Guo et al., 2019). Radix arnebiae oil (RAO), a common TCM formulation, has shown significant therapeutic efficacy in burn treatment. In a rat burn model, topical application of RAO at a dose of 0.3 g twice daily was initiated on day 1 post-burn. This treatment regimen significantly enhanced superoxide dismutase activity, reduces malondialdehyde production, downregulates pro-inflammatory cytokines (IL-6, IL-1β, and TNF-α), and promotes the secretion of growth factors (VEGF, EGF, and TGF-β), thereby accelerating epithelial regeneration and scar repair. Its mechanism may involve activation of the TGF-β1/PI3K/Akt pathway (Gao et al., 2023).

Shikonin (SNK), the principal active component of Arnebia euchroma (Royle) Johnst., exhibits anti-inflammatory, antibacterial, and wound-healing properties. SNK suppresses inflammation by inhibiting the NF-κB signaling pathway, reducing the expression of Bax, p-p65, and p-p38, while upregulating Bcl-2. It also activates the Wnt signaling pathway through the upregulation of Wnt4, promoting cell proliferation and epithelial tissue regeneration (Sun et al., 2022). Topical SNK ointment enhances wound healing by activating the PI3K/Akt pathway and protecting deep hair follicles (Wu T. et al., 2022).

Hypertrophic scar formation remains a significant clinical challenge post-burn. SNK regulates the AMPK/mTOR signaling pathway, promoting autophagy and apoptosis in hypertrophic scar-derived fibroblasts (HSFs) (Xie et al., 2015; Zhang et al., 2023). Animal studies demonstrate that sprayed 1 mL of 1.0 μg/mL SNK onto the surface of hypertrophic scars every 2 days improves scar repair. This improvement is mediated by the suppression of p63, keratin 10, α-smooth muscle actin, TGF-β, and type I collagen (Deng et al., 2018). A recent innovation involves a temperature-sensitive hydrogel composed of chitosan-β-glycerophosphate, mesoporous carbon nanospheres, nitric oxide (NO) donor sodium nitroprusside, and SNK (loaded at 800 μg/mL). This hydrogel exhibits broad-spectrum antibacterial activity, releases NO under near-infrared (NIR) laser irradiation to promote angiogenesis, inhibits fibroblast overproliferation, and effectively reduces scars in deep second-degree burns, highlighting its potential as a novel clinical product for scar treatment (Bai et al., 2024).

Burn wounds are prone to bacterial infections, including methicillin-resistant *Staphylococcus aureus* (MRSA), which often form drug-resistant biofilms. SNK-liposomes (SNK concentration 4.6% ± 0.17%), prepared using a film formation method, exhibit sustained release and strong antibacterial activity by disrupting bacterial cell walls and membranes. These liposomes modulate the IκBα/NFκB-p65 signaling pathway, alleviating inflammation and promoting healing in MRSA-infected burn wounds (Shu et al., 2022). Additionally, bio-adhesive nanoparticles (BNP) based on polylactic acid-hyperbranched polyglycerol (PLA-HPG) enhance SNK’s (loaded at 3.6% ± 0.1%) anti-biofilm and wound-healing properties, making SNK/BNP a promising treatment for infected burn wounds (Han et al., 2023).

### Rhubarb

Rhubarb (Da Huang), the dried rhizome and root of *Rheum palmatum* L. is widely used in TCM to eliminate heat-toxins, clear accumulations, promote blood circulation, and facilitate tissue regeneration. Standardized extracts (typically containing 2%–4% total anthraquinones) is frequently employed in burn treatment. Animal studies demonstrate that rhubarb extract (administered at 50 mg/kg/day) enhances the activity of cytochrome oxidase and superoxide dismutase (SOD) in intestinal mucosal epithelial cells of burned rats, reducing mitochondrial oxygen free radical leakage. It also decreases immunoglobulin A (IgA) content in intestinal fluid and alleviates trauma or burn-induced intestinal mucosal damage (Chen et al., 2000). A clinical trial involving 30 severely burned patients revealed that rhubarb (at 30g/day) increases gastrointestinal hormone secretion, restores gastrointestinal motility, and protects the intestinal mucosal barrier (Meng et al., 2011). Furthermore, rhubarb (at 50 mg/kg) mitigates antibiotic-induced dysbiosis by reducing the bactericidal effect on symbiotic bacteria in early sepsis and exerts anti-inflammatory and immune-regulating effects during burn-induced sepsis (Chen et al., 2009; Liu et al., 2019). Recent studies highlight a scaffold composed of cross-linked chitosan and rhubarb-derived charcoal (RCS/SF), which exhibits rapid hemostasis, antibacterial activity, and efficient drug release (rhubarb extract loaded at 20 or 100 mg). This scaffold promotes diabetic wound healing in db/db mice by enhancing neovascularization, collagen deposition, and re-epithelialization within 2 weeks. Additionally, it modulates the AMPK signaling pathway, reducing hepatic lipid accumulation, inflammation, and oxidative stress, underscoring its systemic regulatory role (Wang et al., 2023; Tan et al., 2024).

Emodin, a primary active compound in rhubarb (standardized to >90% purity), enhances fibroblast fibrinolytic activity and migration at concentrations of 30 or 50 μM, facilitating wound healing (Radha et al., 2008). It also promotes type I collagen synthesis in dermal fibroblasts at 1 μM (Song et al., 2021). In animal studies, emodin (applied at (400 μg/mL) accelerates excisional wound healing by stimulating epidermal cell proliferation, capillary generation, and microcirculation, while reducing inflammation via inhibition of the TLR4/NF-κB signaling pathway (Tang et al., 2007). Emodin also alleviates hypertrophic scar formation by inhibiting macrophage polarization (at 10–40 μM), potentially through suppression of the Notch and TGF-β pathways (Sánchez et al., 2020). Additionally, emodin shows therapeutic potential for corneal alkali burns (at 10–20 μM) by suppressing inflammatory cell infiltration and angiogenesis (Kitano et al., 2007; Xueying et al., 2024). To address bacterial infections, a critical factor in wound healing, nano-emodin (N-EMO)-mediated photodynamic therapy (at 40 μg/mL) effectively targets multi-species bacterial biofilms, reducing biofilm formation and virulence factors (Pourhajibagher et al., 2021). A recent study developed a double-network hydrogel incorporating emodin (at a concentration of 0.03%) and chitosan, which significantly promotes blood vessel and collagen regeneration, accelerating wound healing in animal models (Wan et al., 2023).

### Angelica sinensis

Angelica sinensis, the dried root of *Angelica sinensis* (Oliv.) Diels (known as Dang Gui), is widely used in TCM to promote blood circulation, regulate menstruation, alleviate pain, and relieve constipation (Nai et al., 2021). Standardized extracts (often containing ligustilide >0.5%) are clinically employed to treat various skin wounds and accelerate wound healing. Research has shown that Angelica sinensis extract (at 100 μg/mL) promotes the proliferation of human umbilical vein endothelial cells (HUVECs) by modulating the phosphorylation of p38 and JNK 1/2, upregulating VEGF expression, and facilitating angiogenesis (Lam et al., 2008). Additionally, it enhances the proliferation of human dermal fibroblasts and the production of type I collagen, significantly accelerating wound healing in mice (topical application of 2% gel) (Zhao et al., 2012).

The main bioactive constituents of *Angelica sinensis* (Oliv.) Diels include Angelica sinensis polysaccharides, ligustilide, and ferulic acid (FA). FA (typically used at 10–100 μM *in vitro* or 5–100 mg/kg *in vivo*), in particular, exhibits multifunctional properties such as anti-inflammatory, antibacterial, collagen-promoting, angiogenic, and re-epithelialization effects, making it a promising candidate for burn-related wound healing (Li et al., 2021). Yuhong Ointment (YHO), a traditional formulation used for over 600 years to treat skin diseases, contains active constituents such as FA, L-hydroxyproline, chlorogenic acid, and sermanine. These components exert anti-inflammatory and tissue-regenerative effects, demonstrating significant therapeutic efficacy in burns and scalds (Yu et al., 2023).

A recent study developed a biodegradable, multifunctional spray hydrogel containing FA (at 1.0 wt%), which significantly enhances fibroblast proliferation, accelerates infected wound healing, and prevents secondary injuries, highlighting its potential for clinical application (Zhong et al., 2024). In a rabbit corneal alkali burn model, a thermosensitive chitosan-based hydrogel containing FA (1 mg/mL) significantly reduced inflammatory factors and suppressed cell apoptosis, promoting corneal wound healing (Tsai et al., 2016). Furthermore, a multifunctional hydrogel containing FA (at 2% (w/v)) has been shown to inhibit MRSA infection, reduce excessive inflammation, promote angiogenesis, and accelerate wound healing and skin tissue regeneration (Li et al., 2024). A nano-hydrogel composed of FA-grafted chitosan exhibits enhanced antioxidant activity by scavenging ABTS and DPPH free radicals, while effectively inhibiting *Bacillus subtilis*, MRSA, *Escherichia coli*, and *Pseudomonas aeruginosa*, facilitating the healing of infected wounds (Prasathkumar et al., 2024).

### Coptis chinensis

Coptis chinensis (Huang Lian), the dried *rhizome of Coptis chinensis* Franch., is widely used in TCM to clear heat and dampness, purge fire, and detoxify (Wang et al., 2019). Its standardized extracts, which typically contain total alkaloids >10% (including berberine >5%), are commonly incorporated into modern formulations. Huang Lian Jie Du Decoction, a classic TCM formula, exhibits anti-inflammatory, antibacterial, and microcirculation-improving effects, making it effective for treating burns and febrile diseases (Qi et al., 2019). Berberine (BBR), the main active ingredient of *Coptis chinensis* Franch. (standardized to >97% purity), demonstrates potent antibacterial and anti-inflammatory properties (Kong et al., 2022). BBR inhibits the secretion of pro-inflammatory cytokines (e.g., TNF-α and IL-23) by suppressing NF-κB activity, modulating neutrophil migration, and reducing neutrophil aggregation in inflammatory regions, thereby mitigating inflammatory responses (Habtemariam, 2020). Recent studies indicate that BBR (at 100 mg/kg/d) reduces burn-induced gut vascular barrier hyperpermeability by modulating the S100B/caspase-8/β-catenin pathway, potentially involving enteric glial cells (Feng et al., 2024).

To address antibiotic resistance, Sun S. et al. developed an antibiotic-free polysaccharide-based hydrogel dressing (ATB) containing BBR hydrochloride (loaded at 1 mg/mL). This dressing synergistically eliminates bacteria and accelerates wound healing in both *in vitro* and *in vivo* experiments, offering a solution to the overuse of antibiotics (Sun et al., 2024). Additionally, silk fibroin microspheres loaded with berberine (Ber@MPs, BBR loading 40 mg) exhibit strong antibacterial effects against *Staphylococcus aureus* and *Staphylococcus epidermidis*, reduce inflammation, promote fibroblast migration and endothelial cell neovascularization, and significantly accelerate infected wound healing (Sang et al., 2023; Zhang et al., 2024).

Wound healing, a complex biological process critical for tissue repair, is enhanced by BBR-containing cryogels (loading at 2.08%–5.88%), which accelerate granulation tissue formation, epithelial regeneration, and collagen deposition (Dar et al., 2024). Furthermore, nanofiber dressing patches containing BBR (loading at 4.82%–13.69%) inhibit pro-inflammatory factor secretion by M1 macrophages, promote fibroblast proliferation, and exhibit broad-spectrum antimicrobial activity. Animal studies demonstrate that these dressings (applied at 1–2 mg/cm^2^) accelerate full-thickness skin wound healing, shorten healing time, and improve healing quality, highlighting their potential for treating chronic and difficult-to-heal wounds (Wang Q. et al., 2024).

### Phellodendron amurense

Phellodendri Cortex (Huang Bo), the dried bark of *Phellodendron chinense* Schneid. or *Phellodendron amurense* Rupr. as specified in the Chinese Pharmacopoeia, is widely used in TCM to clear heat, remove dampness, purge fire, and detoxify (Sun et al., 2019). Standardized extracts (typically containing berberine >3%, total alkaloids >5%) are commonly used. Research demonstrates that *Phellodendron chinense Schneid.* extract (at 100–500 mg) exerts immunomodulatory effects by inhibiting the production and secretion of key cytokines such as interferon-γ (INF-γ), IL-1, TNF-α, and IL-2, thereby alleviating inflammatory damage (Xian et al., 2011). It also exhibits antioxidant effects by scavenging free radicals and enhances the phagocytic function of monocytes/macrophages, improving nonspecific immunity (Liu et al., 2022). The main bioactive constituents, including phellodendri, berberine, and other alkaloids, display significant antimicrobial activity against pathogens such as *Staphylococcus aureus*, *Streptococcus albus*, *Streptococcus pneumoniae*, *Bacillus subtilis*, and *Pseudomonas aeruginosa* (Chen et al., 2010).

### Sanguisorba officinalis

Sanguisorba officinalis (Di Yu), the dried rhizome of *Sanguisorba officinalis* L., is widely used in TCM to cool blood, stop bleeding, clear heat, detoxify, reduce swelling, and promote wound healing. Standardized extracts (often containing tannins >10%, total flavonoids >2%) are typically employed. Modern research has identified a rich variety of bioactive compounds in Sanguisorba officinalis, including tannins, triterpenoids, flavonoids, and polysaccharides. Pharmacological studies have demonstrated its diverse functions, such as hemostatic, antibacterial, anti-tumor, anti-allergic, anti-inflammatory, and anti-edema effects (Zhou et al., 2021). The antibacterial activity of *Sanguisorba officinalis* L. is primarily attributed to its tannin components, which exhibit inhibitory effects against pathogens such as *Staphylococcus aureus, Pseudomonas aeruginosa, Acinetobacter baumannii, and Streptococcus pneumoniae* (Zhao et al., 2017). Quercetin, a flavonoid present in *Sanguisorba officinalis*, has been identified as a key contributor to its antioxidant effects, which are beneficial for promoting wound healing (Jang et al., 2018). Additionally, the ethanol extract of *Sanguisorba officinalis* extracts (at 2.5–10 g/kg/d) accelerates diabetic wound healing by inhibiting the NF-κB/NLRP3 signaling pathway and facilitating macrophage polarization from the M1 to the M2 phenotype (Song et al., 2023). A purified polysaccharide (SOP) extracted from *Sanguisorba officinalis* L. has shown remarkable efficacy in mouse burn models, significantly accelerating wound contraction and reducing epithelialization time. SOP administration increases levels of IL-1β and VEGF, promoting granulation tissue formation, collagen synthesis, and angiogenesis, thereby expediting wound repair (Zhang et al., 2018).

### Angelica dahurica

Angelica dahurica (Bai Zhi), the dried root of *Angelica dahurica* (Fisch. ex Hoffm.) Benth. & Hook.f., is traditionally used in TCM to dispel wind, eliminate dampness, reduce swelling, and relieve pain. Standardized extracts (typically containing imperatorin >0.5%, total coumarins >2%) are used. Modern pharmacological studies reveal its anti-inflammatory, analgesic, antispasmodic, and antibacterial properties (Zhao et al., 2022). Animal experiments demonstrate that *Angelica dahurica* (Fisch. ex Hoffm.) Benth. & Hook.f. water extract (at 6g//kg/d) modulates macrophage polarization (M1/M2), exerting anti-inflammatory effects and promoting wound healing (Hu et al., 2021). Its combination with *Angelica sinensis* (Oliv.) Diels and *Rheum officinale Baill.* enhances wound healing during inflammatory and proliferative phases (Yang et al., 2017; Chao et al., 2021). Additionally, *Angelica dahurica* (Fisch. ex Hoffm.) Benth. & Hook.f. promotes angiogenesis in HUVECs by upregulating the HIF-1α/PDGF-β pathway and enhancing angiogenic signals such as ERK1/2, Akt, eNOS, and NO production, suggesting its potential for vascular injury-related wounds (Zhang et al., 2017; Guo et al., 2020). Key bioactive compounds include imperatorin, isoimperatorin, and psoralen. Recent studies highlight isorhamnetin (applied at 0.1%–0.5% topically), which alleviates chronic inflammation, promotes epithelial regeneration, and accelerates *Staphylococcus aureus* infected wound healing through its anti-inflammatory, proliferative, and antibacterial properties (Yang et al., 2020; Lakshmanan et al., 2024).

### Natural borneol

Natural borneol, which is derived from the tree *Dryobalanops aromatica* C.F.Gaertn. and standardized to a purity of >96%, is used in TCM for opening the orifices, improving mental alertness, clearing heat, and relieving pain. Research shows that borneol promotes wound healing by mitigating oxidative stress, facilitating neutrophil recruitment, and suppressing inflammatory cytokines (IL-1β, IL-6, and TNF-α) via inhibition of the HIF-1α/NF-κB pathway (Chen et al., 2024). A chitosan-based film containing 1% borneol (QUIBO1) accelerates wound contraction, enhances granulation tissue formation, and improves collagen density (Barreto et al., 2016). Nanofibers incorporating alum and borneol, fabricated via coaxial electrospinning, increase borneol dissolution and wound healing efficacy (Lv et al., 2022). Additionally, a natural antibacterial hydrogel, synthesized through Schiff base cross-linking of carboxymethyl chitosan and dialdehyde dextran grafted with borneol, exhibits strong antibacterial activity against *Escherichia coli* and *Staphylococcus aureus*, excellent cytocompatibility, and targeted delivery potential for localized wound infections (Zhao et al., 2024).

### Frankincense

Frankincense (Chinese name: Ru Xiang), the resin obtained from *Boswellia sacra* Flück*.* and related species and standardized to contain boswellic acids (>30%), is traditionally used in TCM to promote blood circulation, alleviate pain, and eliminate toxins. Its bioactive constituents, primarily pentacyclic triterpenes (e.g., boswellic acids) and volatile oils, have been demonstrated to possess anti-inflammatory, anti-proliferative, analgesic, antioxidant, and antibacterial properties (Morikawa et al., 2017). Boswellic acids (effective at 5–50 μM) promote wound healing by inhibiting oxidative inflammatory markers, enhancing collagen synthesis and angiogenesis, promoting growth factors, and suppressing apoptosis (Pengzong et al., 2019). Clinical studies confirm the efficacy of myrrh and frankincense-based sitz baths in post-episiotomy wound healing (Faraji et al., 2021). Incorporating essential oils (e.g., clove, cinnamon, frankincense) into biodegradable polymer membranes enhances biological activity and protects against degradation, offering a novel approach to wound healing dressings (Borges et al., 2024). ShengFu Oil, a topical TCM formulation, contains standardized extracts of *Scutellaria baicalensis* Georgi, *Boswellia carterii* Birdw., and *Rheum palmatum* L. It has been demonstrated to possess anti-inflammatory, analgesic, and antibacterial properties. It facilitates burn wound healing through the regulation of key biomarkers (β-catenin, Dlk1, COX-2) and concurrent modulation of the inflammatory microenvironment, playing a vital role in the prevention and treatment of oral chemical burns (Han et al., 2017; Yin et al., 2022).

### Myrrh

Myrrh (Chinese name: Mo Yao), the resin obtained from *Commiphora* myrrha (T.Nees) Engl. and standardized to contain volatile oils (>5%), is traditionally used in TCM to disperse blood stasis, alleviate pain, reduce swelling, and promote tissue regeneration.

The therapeutic efficacy and indications of myrrh are highly similar to those of frankincense, often resulting in their combined application in clinical practice. Research results have shown that the topical application of perilla-frankincense-myrrh volatile oil can attenuate the inflammatory response in the early stage of wounds and expedite wound healing in mice (Batiha et al., 2023). Jinchuang ointment, a TCM compound composed of borneol, catechu, frankincense, and myrrh, displays the functions of promoting angiogenesis, cell proliferation, and migration activity, which is conducive to the enhancement of the wound healing process (Ho et al., 2016). Bisacurone is one of the main bioactive compounds in myrrh. Recent research has demonstrated that the topical application of bisacurone gel (0.5%–2% concentration) can effectively diminish oxidative stress and pro-inflammatory cytokines, promote angiogenesis and granulation tissue formation, and remarkably accelerate the healing of wounds in second-degree burn rats (Yan et al., 2023).

## Treatment of burn with TCM preparations

TCM external treatments are a cornerstone of burn management, offering remarkable therapeutic efficacy with minimal side effects. With advancements in modern TCM pharmacology, a variety of external preparations, such as ointments, sprays, powders, tinctures, hydrogels, and nanofibers, have been developed and widely used in clinical practice. These formulations, combined with innovations in biomaterials and tissue engineering, provide effective solutions for wound management. This review systematically summarizes the application of TCM preparations in burn treatment, with a focus on their active ingredients, therapeutic effects and mechanisms (Table 2).

**TABLE 2:** TCM preparations for burn treatment.

Herbs/Active ingredients	Dosage form	Preparations	Therapeutic effects	Mechanism	References
Radix arnebiae Shikonin (SNK)	Oil Spray Ointment Hydrogel Liposome Nanoparticle	Radix arnebiae oil (RAO) Radix arnebiae spray Shikonin (SNK) ointment Temperature-sensitive hydrogel SNK-liposomes SNK/BNP nanoparticles	Anti-inflammatory Antibacterial Promotes epithelial regeneration Promotes angiogenesis Wound healing Scar reduction	Inhibit NF-κB Activate Wnt/PI3K/Akt Modulate TGF-β1/PI3K/Akt AMPK/mTOR pathways	Shu et al. (2022), Wu T. et al. (2022), Han et al. (2023), Liu et al. (2024)
Rhubarb Emodin Rhubarb-derived charcoal	Scaffold Biofilm Hydrogel	Rhubarb charcoal-crosslinked chitosan/silk fibroin sponge scaffold Nano-emodin (N-EMO) biofilms Chitosan-emodin network hydrogel	Antioxidant Anti-inflammatory Promote tissue regeneration Wound healing	Enhance SOD activity Inhibit TLR4/NF-κB Modulate AMPK pathway	Pourhajibagher et al. (2021), Wan et al. (2023), Wang et al. (2023), Tan et al. (2024)
Angelica sinensis Ferulic acid (FA) Angelica polysaccharides Ligustilide	Ointment Hydrogel	Yuhong Ointment (YHO) CSMA-FA/OBSP (CSOB-FA) hydrogel Thermosensitive chitosan-FA hydrogel Bioactive poly(FA) hydroge FA-grafted chitosan nano-hydrogel	Angiogenesis Collagen synthesis Anti-inflammatory Anti-scarring	Upregulate VEGF Modulate p38/JNK pathways Promote fibroblast proliferation	Tsai et al. (2016), Yu et al. (2023), Li et al. (2024), Prasathkumar et al. (2024), Zhong et al. (2024)
Coptis chinensis Berberine (BBR)	Decoction Hydrogel Cryogel Microsphere Nanofiber	Huang Lian Jie Du Decoction Polysaccharide-based hydrogel with BBR BBR-containing cryogels Berberine-loaded silk fibroin microspheres BBR nanofiber dressing patches	Antibacterial Anti-inflammatory Promote fibroblast migration Enhance neovascularization Wound healing	Inhibit NF-κB, Modulates S100B/caspase-8/β-catenin ↑Granulation tissue formation, collagen deposition and epithelial regeneration	Qi et al. (2019), Sang et al. (2023), Dar et al. (2024), Sun et al. (2024), Wang Q. et al. (2024)
Phellodendron amurense Coptis chinensis Scutellaria	Ointment	Moist Exposed Burn Ointment (MEBO)	Antibacterial Analgesi Promote granulation tissue formation Activate epidermal stem cells	↑VEGF/bFGF Activate PI3K-Akt-mTOR pathway Induce the autophagy process	Mabvuure et al. (2020), Zheng et al. (2020)
Phellodendron amurense Coptis chinensis Rhubarb	Powder	Sanhuang powder	Anti-inflammatory Heat-clearing Detoxifying	↓IL-8/GM-CSF	Wu et al. (2021)
Phellodendron amurense Cinnabar, Safflower	Tincture	Qi Wei Anti-burn Tincture	Anti-inflammatory Antioxidant Liver protection	↑TGF-β1, FGF-2 ↓TNF-α, IL-1β, IL-6 ↓ROS reduction	(Wang S. et al., 2024b)
Borneol	Film Hydrogel Nanofiber	Borneol-chitosan film Schiff base-crosslinked hydrogel Alum/borneol coaxial nanofibers	Anti-inflammatory Antibacterial activity Wound healing	Inhibit HIF-1α/NF-κB Promote granulation Improve collagen density	Barreto et al. (2016), Lv et al. (2022), Zhao et al. (2024)
Aloe vera, Borneol Musk, Mint	Gel	Aloe vera gel	Reduce itching and pain Enhance re-epithelialization	Stimulate fibroblast and keratinocyte proliferation	Mahboub et al. (2021)
Scutellaria baicalensis Frankincense, Rhubarb	Oil	ShengFu Oil	Anti-inflammatory Wound healing	Regulate β-catenin/Dlk1/COX-2	Yin et al. (2022)
Borneol, Catechu, Frankincense, Myrrh	Ointment	Jinchuang ointment	Stimulate angiogenesis Promote cell proliferation Enhance cell migration	Angiogenic activity Wound healing promotion	Ho et al. (2016)
Bisacurone	Gel	Chitosan-based bisacurone gel	Anti-inflammatory Oxidative stress reduction Enhance angiogenesis	↓Pro-inflammatory cytokines ↓MDA, NO; ↑SOD, glutathione ↑Growth factors	Yan et al. (2023)
Curcumin	Hydrogel Nanofiber	Curcumin-loaded magnesium polyphenol network (Cur-Mg@PP) hydrogel mPEG-CUR loaded PVA/CS-g-PNVIS nanofibers	Antimicrobial Antioxidant, Anti-inflammatory Analgesic, Angiogenesis Tissue regeneration	Enhance biocompatibility Enable electrospinning process Structure mimics ECM Moist wound environment maintenance	Gong et al. (2023); Shaabani et al. (2023)
Epigallocatechin gallate	Injectable hydrogel	GelMA/HA-E/Ag@MOF composite hydrogel	Antibacterial/Anti-inflammatory Accelerated wound closure Angiogenesis promotion	Macrophage polarization (M1→M2) Activation Noncanonical Wnt pathway	Xiong et al. (2022)
Asiaticoside	Injectable hydrogel	rColMA/QCSG/LIP@AS/Ag@MOF (RQLAg) hydrogel	Antibacterial Anti-inflammatory Accelerates wound healing	Activate M1 macrophages Promote angiogenesis Enhance cell migration	Feng et al. (2023)
Alginate	Core-shell nanofiber	Asiaticoside-loaded nanofibers	Antibacterial/Anti-inflammatory Angiogenesis promotion	↑VEGF, CD31 expression ↓TNF-α, IL-6 Improve cell proliferation	Zhu et al. (2016)
Lavender active compound	Electrospun nanofiber	Alginate-lavender essential oil nanofibers	Antibacterial,Anti-inflammatory UVB burn protection Prevent erythema formation Promote tissue regeneration	Moist wound environment Biocompatibility Wound exudate management	Hajiali et al. (2016)
Bakuchiol	Nanofibrous electrospun scaffold	Bakuchiol nanoemulsion-loaded gelatin scaffold	Antioxidant, Analgesic Enhanced wound healing Antibacterial, Anti-inflammatory	Enhance BAK stability Controll drug release Uniform biomarker distribution	Kaur et al. (2024)

### Ointments

Ointments are a common dosage form for burn treatment, offering excellent adhesiveness and direct application to wounds. They prevent external stimuli and bacterial infections while providing anti-inflammatory, analgesic, and tissue-repairing effects. Moist Exposed Burn Ointment (MEBO), a patented TCM formulation, accelerates wound healing, exhibits antibacterial properties, and alleviates pain (Mabvuure et al., 2020). MEBO enhances granulation tissue formation, promotes the production of VEGF and bFGF, and activates epidermal stem cells (El-Hadidy et al., 2014; Tang et al., 2014). It also facilitates diabetic ulcer healing through autophagy and the PI3K-Akt-mTOR signaling pathway (Zheng et al., 2020). Aloe vera burn cream stimulates fibroblast and keratinocyte proliferation, significantly improving re-epithelialization rates and outperforming 1% sulfadiazine silver cream in treating second-degree burns (Teplicki et al., 2018; Mahboub et al., 2021).

### Sprays

Sprays offer a convenient application method, reducing pain during drug administration and making them ideal for large-area burns. They form a breathable, elastic film on the wound surface, promoting granulation tissue growth. A spray formulation containing the extracts of *Arnebia euchroma* (Royle) Johnst.), *Taraxacum mongolicum* Hand.-Mazz., *Phellodendron chinense* Schneid. and borneol rapidly forms a protective film within 3–5 min. This film effectively shields wounds from contamination and infection while accelerating eschar formation (Liu et al., 2024). Autologous cell spray grafting, an innovative approach, uses a suspension of the patient’s skin cells to treat deep burns, significantly enhancing re-epithelialization and wound healing (Esteban-Vives et al., 2016; Shree and Vagga, 2022).

### Powders

Powders are simple to prepare and effectively absorb necrotic tissue from burn wounds. However, they may cause excessive crust formation and contamination risks. Sanhuang Powder, a classic TCM formula composed of *Rheum palmatum* L., *Phellodendron chinense* Schneid., and *Coptis chinensis* Franch., is used to clear heat and resolve toxins. Modern studies indicate that it also reduces the levels of pro-inflammatory cytokines, including IL-8 and GM-CSF (Wu et al., 2021). Jinhuang powder, a classic TCM surgical preparation, promotes fibroblast proliferation and migration via the Wnt/β-catenin signaling pathway, effectively treating diabetic foot wounds when combined with MEBO (Zhan et al., 2021; Wu M. et al., 2022).

### Tinctures

Tinctures, which are herbal extracts dissolved in ethanol, facilitate easy monitoring of wounds; however, their irritant properties limit their application to first-degree burns. The Qi Wei Anti-burn Tincture, formulated with *Phellodendron chinense* C.K.Schneid., *Melaleuca phoenicea* (Lindl.) Craven and synthetic borneol, has been demonstrated to upregulate the expression of growth factors TGF-β1 and FGF-2, while downregulating the levels of pro-inflammatory mediators (TNF-α, IL-1β, IL-6) and reactive oxygen species in the livers of burned mice (Dinda et al., 2015; Wang S. et al., 2024).

### Hydrogels

Hydrogels are highly promising for burn treatment due to their ability to adhere to uneven wound surfaces, inhibit bacterial growth, and reduce pain during dressing changes (Stoica et al., 2020). Multifunctional hydrogels derived from TCM active components offer antibacterial, anti-inflammatory, hemostatic, and tissue-regenerative properties (Shu et al., 2021). For example, a magnesium polyphenol network (Cur-Mg@PP) hydrogel loaded with curcumin demonstrates excellent therapeutic effects in pain relief, anti-inflammation, angiogenesis, and tissue regeneration (Gong et al., 2023). Another hydrogel loaded with epigallocatechin gallate exhibits dual antibacterial and anti-inflammatory properties, accelerating wound healing via the non-classical Wnt signaling pathway (Xiong et al., 2022). Additionally, a liposome-based hydrogel containing asiaticoside and superfine silver nanoparticles promotes cell migration, angiogenesis, and M1 macrophage polarization, effectively treating bacterial-infected burn wounds (Feng et al., 2023).

### Nanofibers

Nanofibers loaded with bioactive compounds like curcumin and quercetin mimic the extracellular collagen matrix, supporting cell growth and accelerating burn wound healing (Shaabani et al., 2023). Asiaticoside-loaded nanofibers exhibit fast drug release and anti-inflammatory effects, significantly promoting healing in deep partial-thickness burns (Zhu et al., 2016). Alginate-lavender nanofibers possess antibacterial and anti-inflammatory properties, effectively treating burns by inhibiting *Staphylococcus aureus* growth and reducing inflammation in fibroblasts (Hajiali et al., 2016). Recent research shows that bakuchiol nanoemulsion-loaded gelatin scaffold exhibit significant analgesic, anti-inflammatory and wound healing promoting effects, and have potential application value in the treatment of burn wounds (Kaur et al., 2024).

### Treatment of burn with TCM auxiliary therapies

In addition to TCM drugs and preparations, the TCM system includes unique therapies such as acupuncture, cupping, gua sha, tuina, and aromatherapy. These therapies complement conventional treatments, enhancing burn recovery and improving patients’ quality of life. Acupuncture, a cornerstone of TCM, plays a vital role in burn management. It alleviates pain, modulates inflammatory responses, promotes epithelialization and angiogenesis, and accelerates wound healing. A case study involving 1,008 burn patients demonstrated that acupuncture significantly improves wound healing outcomes in medical, economic, and biopsychosocial aspects (Loskotova and Loskotova, 2017). Acupoint stimulation therapy, a key form of acupuncture, modulates the neuroendocrine system by targeting specific acupoints such as Quchi, Hegu, Taichong, Xuehai, Sanyinjiao, and Zhiyang, reducing pain and inflammation. Electroacupuncture, which combines traditional acupuncture with electrical stimulation, enhances blood circulation, improves nerve conduction, reduces edema, and promotes wound healing. A randomized controlled trial showed that electroacupuncture at the bilateral Dingchuan acupoint improves lung function and diaphragm activity in patients with inhalation burns (Ali et al., 2022). Auricular therapy, targeting ear acupoints like Shenmen and Subcortical, effectively reduces pain, itching, and sleep disturbances in burn patients (Chen et al., 2021). Cupping induces local congestion through negative pressure, promoting blood circulation and lymphatic drainage. This process accelerates toxin elimination and generates anti-inflammatory, analgesic, and wound-healing effects. Before treatment, the burn area must be thoroughly cleaned, and key acupoints such as DU14 (Dazhui), LI11 (Quchi), and ST36 (Zusanli) are selected. Cupping can be performed using fire cups, air cups, or electric cups, followed by skin cleaning and medicinal cream application (Al-Bedah et al., 2019). Gua sha and tuina improve local blood circulation, enhance metabolic processes, relieve inflammation and edema, reduce pain, and promote wound healing. These manual therapies are particularly effective in managing burn-related discomfort and accelerating recovery (Xie et al., 2022). Aromatherapy utilizes plant essential oils extracted from flowers, leaves, and fruits to promote physical and mental wellbeing. It helps relax the nervous system, reduce stress, facilitate deep sleep, and alleviate the physical and mental stress caused by burns (Lee et al., 2021).

### Combined treatment of burn with TCM and modern medicine

The integration of TCM and modern medicine represents a pivotal strategy in burn treatment, combining the strengths of both systems to optimize patient outcomes. Western medicine excels in rapid infection control, pain relief, and body temperature regulation, while TCM offers a holistic approach, minimal side effects, and favorable conditions for recovery. Together, they provide complementary benefits that address the multifaceted challenges of burn management. Modern medicine’s dry therapy facilitates convenient wound observation and rapid healing by drying the wound and promoting scabbing, often followed by surgical or other reparative interventions. In contrast, TCM’s moist therapy emphasizes moist wound repair, promotes physiological regeneration, and reduces scar formation. Additionally, traditional TCM therapies such as acupuncture and cupping play a distinctive role in alleviating pain, enhancing local blood circulation, and improving overall recovery. The research and development of TCM preparations further expand treatment options through the integration of TCM and modern medicine. However, several challenges remain. Firstly, the multifaceted components and diverse mechanisms of TCM make it difficult to fully elucidate its efficacy using modern medical standards. Secondly, inconsistent quality control standards in TCM may lead to batch-to-batch variations, affecting reproducibility and reliability. Finally, variations in treatment methods and medication practices between TCM and Western medicine necessitate enhanced communication and collaboration to bridge gaps and optimize integrated care.

## Conclusion

TCM has a long history and a well-established theoretical system for treating burn injuries, providing a robust foundation for modern scientific evaluation. As TCM modernization advances, the active ingredients and molecular mechanisms underlying its efficacy in burn treatment have been increasingly elucidated. This review explores the approaches and research progress in TCM for burn management, summarizing current TCM drugs, external preparations, adjunctive therapies, and their potential mechanisms. Regarding safety concerns, topical TCM applications for burn treatment generally demonstrate favorable safety profiles with minimal systemic side effects, owing to their localized administration and natural origins. However, vigilance remains essential as certain herbal components may cause local skin reactions, allergic responses, or interact with conventional therapies. High-dose applications of specific active ingredients, particularly alkaloids and anthraquinones, warrant careful consideration due to potential cytotoxic effects at elevated concentrations. Furthermore, the integration of TCM with modern burn therapies necessitates attention to potential pharmacological interactions, especially when combining herbal preparations with systemic medications.

Future research should prioritize comprehensive safety assessments, including long-term toxicity studies and drug interaction profiling, to establish evidence-based guidelines for safe clinical application. Most TCM burn treatments remain limited to animal models, highlighting the need for randomized, double-blind, placebo-controlled clinical trials in human patients to validate their efficacy and safety. Furthermore, the diversification of TCM external dosage forms, driven by advancements in pharmaceutical technology, presents both challenges and opportunities. Future research should focus on enhancing TCM preparation development, establishing systematic quality control systems, and improving therapeutic efficacy and safety. These efforts are essential for integrating TCM into mainstream burn treatment protocols and optimizing patient outcomes.

## Glossary

TCM: Traditional Chinese Medicine
TGF-β: transforming growth factor-β
PDGF: platelet-derived growth factor
EGF: epidermal growth factor
FGF: fibroblast growth factor
IL-8: interleukin-8
IL-6: interleukin-6
IL-1β: interleukin-8
IL-2: interleukin-8
IL-23: interleukin-8
TNF-α: tumor necrosis factor-alpha
VEGF: vascular endothelial growth factor
ECM: extracellular matrix
MMPs: matrix metalloproteinases
RAO: Radix arnebiae oil
SNK: shikonin
HSFs: hypertrophic scar-derived fibroblasts
MRSA: methicillin-resistant *Staphylococcus aureus*
SOD: superoxide dismutase
IgA: immunoglobulin A
HUVECs: human umbilical vein endothelial cells
FA: ferulic acid
BBR: Berberine
MEBO: Moist Exposed Burn Ointment
NO: nitric oxide
NIR: near-infrared
BNP: bio-adhesive nanoparticles
PLA-HPG: polylactic acid-hyperbranched polyglycerol
RCS/SF: scaffold composed of cross-linked chitosan and rhubarb-derived charcoal
N-EMO: nano-emodin
SDT: photodynamic therapy
YHO: Yuhong Ointment
ABTS: 2,2′-azino-bis-(3-ethylbenzthiazoline-6-sulfonic acid)
DPPH: 1,1-Diphenyl-2-picrylhydrazyl radical
ATB: antibiotic-free polysaccharide-based hydrogel dressing
Ber@MPs: silk fibroin microspheres loaded with berberine
SOP: polysaccharide
QUIBO1: chitosan-based film containing 1% borneol
Cur-Mg@PP: magnesium polyphenol network
